# Persistent Post-ablative Erythema After Carbon Dioxide Laser Resurfacing Producing a Pseudoatrophic Appearance: Impact on Clinical Assessment of Skin Surface Topography

**DOI:** 10.7759/cureus.108176

**Published:** 2026-05-03

**Authors:** Yasir Radhi, Ali Almamoori

**Affiliations:** 1 Department of Dermatology, Al-Shaheed Al-Sadr General Hospital, Baghdad, IRQ; 2 Department of Dermatology, Baghdad Teaching Hospital, Baghdad, IRQ

**Keywords:** atrophic acne scars, optical masking, persistent erythema, post-inflammatory erythema, vascular laser (577-nm yellow laser)

## Abstract

Persistent erythema following ablative carbon dioxide (CO_2_) laser resurfacing is attributed to prolonged post-inflammatory vascular proliferation and may alter optical skin properties during the remodeling phase. We report a case of a 39-year-old woman who presented with diffuse erythema three months after CO_2_ resurfacing for acne scars. The treated areas demonstrated a smooth, shiny appearance with reduced visibility of follicular ostia, raising concern for atrophic change. The patient underwent four sessions of 577-nm yellow laser therapy (FlexSys®; GME German Medical Engineering GmbH, Erlangen, Germany) at one-month intervals. Progressive reduction of erythema was observed, with significant improvement in vascular parameters on bilateral VISIA® analysis (Canfield Scientific, Inc., Parsippany, USA). As redness resolved, follicular ostia and baseline microrelief became more apparent. Decreased texture and pore percentiles were interpreted as enhanced visibility of the underlying skin's surface features rather than deterioration of the skin's quality. Findings suggest that persistent post-ablative erythema may transiently produce a pseudoatrophic appearance through vascular optical masking. Awareness of this phenomenon may prevent misinterpretation of early post-resurfacing outcomes.

## Introduction

Ablative fractional carbon dioxide (CO_2_) laser resurfacing is a well-established and highly effective modality for the treatment of acne scars, photoaging, and skin texture irregularities [1]. However, prolonged post-procedural erythema occurs in a substantial proportion of patients, particularly after deeper or repeated treatments, and can persist for several months [2,3]. This erythema is believed to result from sustained vascular proliferation and delayed regression of neoangiogenesis during the dermal remodeling phase, sometimes leading to patient dissatisfaction and reduced quality of life.

Advanced imaging systems such as VISIA® complexion analysis (Canfield Scientific, Inc., Parsippany, USA) are increasingly used in dermatology to objectively quantify the skin's surface characteristics, including erythema, texture, and pigmentation. Nevertheless, these optical-based systems can be significantly influenced by the underlying vascular changes, pigmentation, and lighting conditions. In erythematous or recently resurfaced skin, this may result in overestimation of surface irregularities and misinterpretation of true dermal topography [4].

Distinguishing true dermal atrophy from a pseudoatrophic appearance caused by persistent erythema is clinically important. The latter represents a transient, optically perceived phenomenon rather than irreversible structural dermal loss. Misinterpretation may lead to unnecessary patient anxiety or inappropriate additional interventions.

Herein, we present a case of a 39-year-old woman who developed persistent diffuse erythema three months after fractional ablative CO_2_ laser resurfacing for acne scars. This erythema produced a pseudoatrophic appearance on VISIA® imaging that gradually improved with time and targeted vascular laser therapy. Through this report, we highlight the potential limitations of relying solely on optical imaging systems in post-procedural skin and underscore the critical role of clinicopathological correlation in accurate diagnosis and management.

## Case presentation

A 39-year-old woman with Fitzpatrick skin phototype III presented with persistent facial erythema three months after undergoing ablative CO_2_ laser resurfacing for acne scarring confined to her bilateral cheek areas.

Clinical examination revealed no objective evidence of true dermal atrophy. The skin maintained normal thickness and structural integrity, with no palpable depressions, textural irregularity on palpation, or shadowing on side-lighting. Dermoscopic evaluation showed preserved follicular openings and normal skin markings.

**Figure 1:**
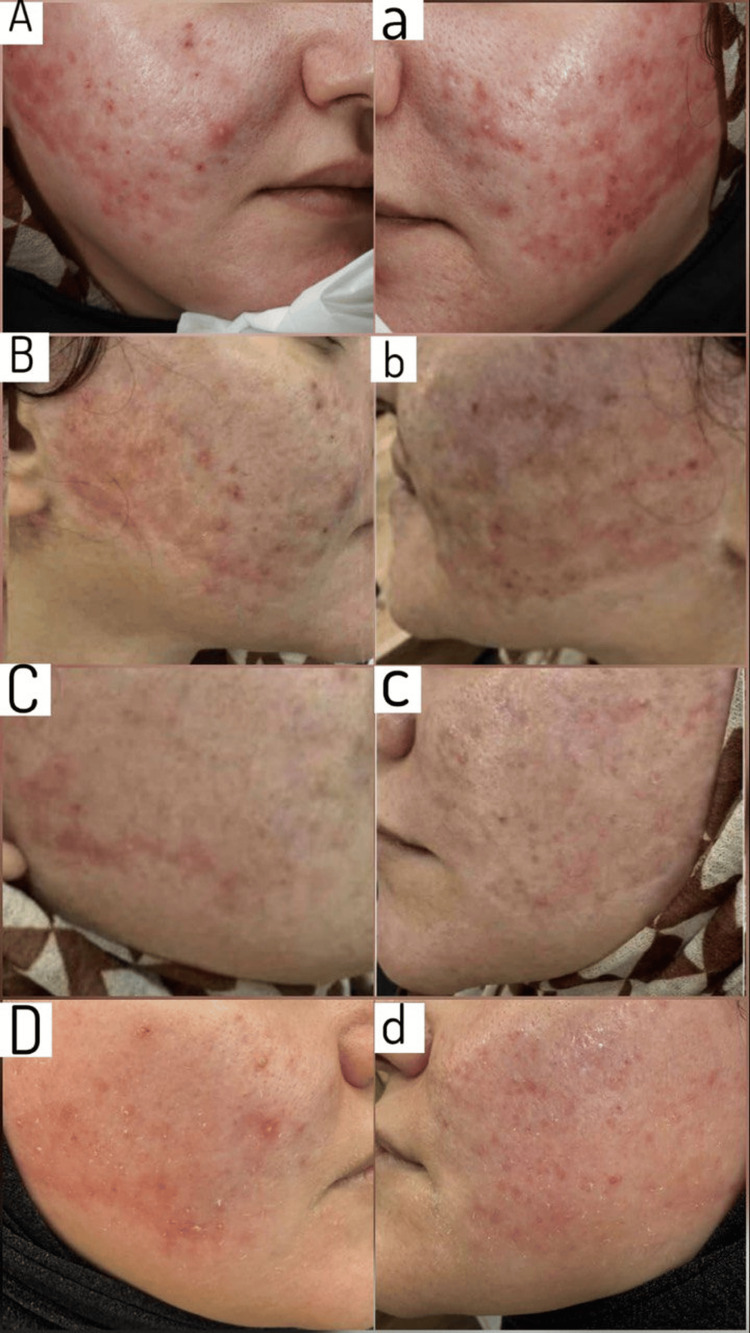
Sequential clinical photographs demonstrating evolution of persistent post-ablative erythema following 577-nm yellow laser treatment. Uppercase panels (A-D) represent the right facial side; lowercase panels (a-d) represent the left facial side. (A, a) Baseline presentation three months after ablative carbon dioxide (CO_2_) laser resurfacing shows diffuse erythema with a relatively smooth surface over the treated cheek areas. (B, b) After the first session of the 577-nm yellow laser, a partial reduction in erythema is observed. (C, c) After completion of four treatment sessions, a further decrease in vascular redness and improved surface definition are observed. (D, d) The final follow-up, nine months after the last yellow laser session, demonstrates sustained reduction of erythema and stabilization of skin appearance.

Importantly, the sharp color contrast between the persistent bright erythematous areas and the adjacent normal-appearing skin created a strong optical illusion of surface depression and atrophy. This visual effect made the skin appear atrophic on standard clinical photography (Figures 1A-1a).

Imaging using the VISIA® complexion analysis system initially demonstrated findings that mimicked dermal atrophy: markedly elevated erythema index, reduced apparent pore count, and smoother skin texture with fewer surface irregularities in the affected areas. These features produced a pseudoatrophic pattern on the VISIA® report.

Given the predominance of vascular findings, treatment with a 577-nm yellow laser (FlexSys®; GME German Medical Engineering GmbH, Erlangen, Germany) was initiated using a fluence of 23 J/cm², a pulse duration of 20 ms, a 1 × 1 cm spot size, 50% coverage, and a single-pass technique. Four treatment sessions were performed at one-month intervals. No additional resurfacing procedures or adjunctive vascular therapies were administered during this treatment. Standardized clinical photography and bilateral VISIA® complexion analysis were obtained prior to initiation of yellow laser therapy. Final evaluation was conducted four months after completion of the last treatment session to ensure stabilization of vascular and inflammatory responses.

Sequential 577-nm yellow laser treatment resulted in progressive reduction of persistent post-ablative erythema. Clinically, diffuse redness diminished gradually over the treatment course. As the erythema areas resolved, follicular ostia became more readily appreciable, and normal skin surface contour became more defined. The patient reported improvement in the burning sensation and cutaneous sensitivity. No dyspigmentation, purpura, scarring, or other adverse events were observed.

Bilateral VISIA® analysis demonstrated marked improvement in vascular parameters (Table 1). Mean red area percentile increased from 9% at baseline to 40.5% at final follow-up, representing a 31.5-point improvement. The right facial side demonstrated greater vascular normalization (9% to 63%) compared to the left (9% to 18%). In contrast, the mean texture percentile decreased from 29% to 10%, and the mean pore percentile decreased from 22.5% to 14.5%. These changes occurred in parallel with the resolution of erythema. The increased detection of texture features and pore parameters following vascular normalization was interpreted as enhanced visibility of baseline skin microrelief and follicular ostia, rather than deterioration in skin quality. Clinical appearance remained stable at fourth-month follow-up after the final yellow laser session.

**Table 1:** Bilateral VISIA® complexion analysis at baseline versus final follow-up.

Parameter	Baseline Right	Final Right	Baseline Left	Final Left
Red area (percentile)	9%	63%	9%	18%
Texture (percentile)	30%	10%	28%	10%
Pores (percentile)	22%	14%	23%	15%

## Discussion

Persistent erythema following ablative CO_2_ resurfacing is commonly attributed to prolonged vascular proliferation and delayed regression of post-injury neoangiogenesis during dermal remodeling [1-4].

Increased dermal vascularity alters optical skin properties through hemoglobin-related light absorption and scattering, producing a more uniform and visually smoother skin appearance during the early post-procedural phase. This vascular predominance may obscure accurate assessment of skin surface topography and may lead to misinterpretation of early post-resurfacing skin texture, sometimes creating a clinical impression of skin atrophy [5,6].

In the present case, persistent erythema was associated with a smooth and shiny surface appearance and reduced visibility of follicular ostia. Progressive resolution of erythema following vascular laser treatment resulted in reappearance of follicular openings and increased visibility of skin surface features. This clinical evolution suggested that the initial appearance reflected vascular optical masking rather than true dermal atrophy, representing transient pseudoatrophic changes during the erythematous phase.

Objective VISIA® analysis supported this interpretation [7,8], demonstrating increased visibility of follicular ostia and higher skin texture parameters following the reduction of erythema. Rather than representing deterioration of skin quality, these findings were interpreted as restoration of normal skin surface topography that had previously been masked by vascular erythema. Mild residual dermal edema during the erythematous phase may further contribute to a smooth surface appearance by reducing surface shadowing and masking superficial contour irregularities.

Treatment with a 577-nm yellow laser facilitated the reduction of persistent erythema and improvement of the associated burning sensation and skin sensitivity [9,10]. In addition to vascular targeting, vascular lasers may indirectly support dermal recovery by reducing inflammation and improving the local healing environment, allowing for a more reliable evaluation of post-resurfacing outcomes once vascular activity normalizes.

From a clinical perspective, these findings highlight the potential for persistent vascular erythema to influence perceived skin surface topography following ablative resurfacing. Premature assessment during the erythematous phase may lead to misinterpretation of surface smoothness as dermal atrophy or, conversely, apparent texture “worsening” after resolution of the redness, which may be interpreted as a procedural failure. Recognition of this phenomenon may assist clinicians in appropriately timing post-resurfacing evaluation and in counseling patients regarding expected visual changes during recovery.

## Conclusions

The present case suggests an association between persistent post-ablative erythema and a transient pseudoatrophic clinical appearance during the remodeling phase, potentially mediated through vascular-related optical masking of baseline skin microrelief and follicular structures. While a causal relationship cannot be established from a single case report, the sequential clinical and objective VISIA® imaging findings support this as a plausible mechanistic hypothesis. As vascular activity normalizes, underlying surface features may become more visible, and clinicians should be aware that this progressive unmasking may be misinterpreted as textural deterioration if assessments are made prematurely during the erythematous phase. Appropriate timing of post-procedural evaluation and proactive patient counseling regarding expected visual changes during recovery remain important clinical considerations. Further prospective studies incorporating objective surface topography measurement and histological assessment are warranted to better define the relationship between vascular dynamics and perceived skin texture following ablative resurfacing and to validate the proposed mechanism at an evidentiary level beyond single-case observation.
